# Plasma lipidome and risk of atrial fibrillation: results from the PREDIMED trial

**DOI:** 10.1007/s13105-023-00958-0

**Published:** 2023-04-01

**Authors:** Estefania Toledo, Clemens Wittenbecher, Cristina Razquin, Miguel Ruiz-Canela, Clary B. Clish, Liming Liang, Alvaro Alonso, Pablo Hernández-Alonso, Nerea Becerra-Tomás, Fernando Arós-Borau, Dolores Corella, Emilio Ros, Ramón Estruch, Antonio García-Rodríguez, Montserrat Fitó, José Lapetra, Miquel Fiol, Ángel M. Alonso-Gomez, Luis Serra-Majem, Amy Deik, Jordi Salas-Salvadó, Frank B. Hu, Miguel A. Martínez-González

**Affiliations:** 1 Department of Preventive Medicine and Public Health, Edificio de Investigación, University of Navarra, Planta 2, Calle Irunlarrea 1, 31008 Pamplona, Spain; 2 IdiSNA, Navarra Institute for Health Research, Pamplona, Spain; 3 CIBER Fisiopatología de La Obesidad Y Nutrición (CIBEROBN), Instituto de Salud Carlos III (ISCIII), Madrid, Spain; 4 SciLifeLab & Division of Food and Nutrition Science, Department of Life Sciences, Chalmers University of Technology, Gothenburg, Sweden; 5 Department of Nutrition, Harvard T.H. Chan School of Public Health, Boston, MA USA; 6 Department of Molecular Epidemiology, German Institute of Human Nutrition Potsdam-Rehbruecke, Nuthetal, Germany; 7 Broad Institute of MIT and Harvard, Cambridge, MA USA; 8 Department of Biostatistics, Harvard TH Chan School of Public Health, Boston, MA USA; 9 Department of Epidemiology, Harvard TH Chan School of Public Health, Boston, MA USA; 10 Department of Epidemiology, Rollins School of Public Health, Emory University, Atlanta, GA USA; 11 Departament de Bioquímica I Biotecnologia, Universitat Rovira I Virgili, Unitat de Nutrició Humana, Reus, Spain; 12 Institut d’Investigació Sanitària Pere Virgili (IISPV), Hospital Universitari San Joan de Reus, Reus, Spain; 13 Unidad de Gestión Clínica de Endocrinología Y Nutrición del Hospital Virgen de La Victoria, Instituto de Investigación Biomédica de Málaga (IBIMA), Málaga, Spain; 14 Department of Preventive Medicine and Public Health, School of Medicine, University of Valencia, Valencia, Spain; 15 Bioaraba Health Research Institute Osakidetza Basque Health Service, Araba University Hospital University of the Basque Country UPV/EHU, Vitoria-Gasteiz, Spain; 16 Department of Endocrinology & Nutrition, Hospital Clinic, Barcelona, Spain; 17 Department of Internal Medicine, Institut d’Investigacions Biomèdiques August Pi I Sunyer (IDIBAPS), Hospital Clinic, University of Barcelona, Barcelona, Spain; 18 Department of Public Health and Psychiatry, University of Malaga, Málaga, Spain; 19 Cardiovascular Risk and Nutrition Group, Hospital del Mar Medical Research Institute (IMIM), Barcelona, Spain; 20 Department of Family Medicine, Research Unit, Distrito Sanitario Atención Primaria Sevilla, 41013 Seville, Spain; 21 IdISBa. Health Research Institute of the Balearis Islands, Palma, Spain; 22 Research Institute of Biomedical and Health Sciences, University of Las Palmas de Gran Canaria, & Centro Hospitalario Universitario Insular Materno Infantil (CHUIMI) del Servicio Canario de Salud, Gobierno de Canarias, Las Palmas de Gran Canaria, Spain; 23 Channing Division for Network Medicine, Department of Medicine, Brigham and Women’s Hospital and Harvard Medical School, Boston, MA USA

**Keywords:** Atrial fibrillation, Metabolomics, Lipidomics, Nested case–control study, Dietary intervention

## Abstract

The potential role of the lipidome in atrial fibrillation (AF) development is still widely unknown. We aimed to assess the association between lipidome profiles of the Prevención con Dieta Mediterránea (PREDIMED) trial participants and incidence of AF. We conducted a nested case–control study (512 incident centrally adjudicated AF cases and 735 controls matched by age, sex, and center). Baseline plasma lipids were profiled using a Nexera X2 U-HPLC system coupled to an Exactive Plus orbitrap mass spectrometer. We estimated the association between 216 individual lipids and AF using multivariable conditional logistic regression and adjusted the *p* values for multiple testing. We also examined the joint association of lipid clusters with AF incidence. Hitherto, we estimated the lipidomics network, used machine learning to select important network-clusters and AF-predictive lipid patterns, and summarized the joint association of these lipid patterns weighted scores. Finally, we addressed the possible interaction by the randomized dietary intervention.

Forty-one individual lipids were associated with AF at the nominal level (*p* < 0.05), but no longer after adjustment for multiple-testing. However, the network-based score identified with a robust data-driven lipid network showed a multivariable-adjusted OR_per+1SD_ of 1.32 (95% confidence interval: 1.16–1.51; *p* < 0.001). The score included PC plasmalogens and PE plasmalogens, palmitoyl-EA, cholesterol, CE 16:0, PC 36:4;O, and TG 53:3. No interaction with the dietary intervention was found. A multilipid score, primarily made up of plasmalogens, was associated with an increased risk of AF. Future studies are needed to get further insights into the lipidome role on AF.

Current Controlled Trials number, ISRCTN35739639.

**Supplementary Information:**

The online version contains supplementary material available at 10.1007/s13105-023-00958-0.

## Introduction

Atrial fibrillation (AF) is an emerging cardiovascular epidemic representing the most common clinically meaningful arrhythmia. Worldwide, around 5 million new diagnoses are made every year [6]. AF is associated with a lower quality of life, higher subsequent risk of stroke, heart failure (HF), and mortality [16]. AF-associated burden measured as disability-adjusted life-years increased by 19% from 1990 to 2010 [6], and available treatments convey relevant risks [12]. Thus, the best option is prevention and, consequently, a deeper understanding of its pathophysiology and etiological determinants is needed.

Metabolomic profiling techniques can help provide more comprehensive insights into AF causes, especially in relationship with nutritional exposures, as this condition has been previously modulated by dietary interventions and exposures [25]. Metabolic profiles reflect gene and protein functional activity and are simultaneously sensitive to lifestyle, in addition to results of gene-regulated metabolic pathways [5].

Although conceptually appealing, prospective studies on metabolomics and AF are scarce [1, 13, 17, 18, 33]. Two studies have been conducted within two observational cohorts, the Atherosclerosis Risk in Communities (ARIC) Study and the Framingham Heart Study, reporting no significantly AF-associated single metabolites after multiple testing adjustment [1, 17]. On the other hand, in the Cardiovascular Health Study, ceramides and sphingomyelins with palmitic acid—Cer-16 and SM-16—were associated with an increased risk of AF, whereas ceramides and sphingomyelins with very-long-chain saturated fatty acids—Cer-20, Cer-22 and Cer-24 and SM-20, SM-22, and SM-24—were inversely associated with AF risk [13]. Additionally, in the ADVANCE trial, GM3(d18:1/41:1) and GM3(d18:1/18:0) were inversely associated with the risk of developing AF [33]. Interestingly, in the latter study, the authors also performed a cluster analyses in which a cluster containing GM3(d18:1/24:1) and closest related lipid species was relevant to prediction of future AF. Recently, three Swedish cohorts on this topic reported that only sphingomyelin (28:1) was significantly and inversely associated with AF risk in a secondary analysis after multiple testing adjustment [18]. Beyond observational studies, analyses of nutritional intervention randomized trials can elucidate the effects of diet on metabolites and their relationships with the subsequent risk of new-onset AF. This approach can provide stronger causal inferences with the potential to be translated to preventive strategies. In fact, some randomized trials have shown sizable effects of nutrition-related interventions on the risk of AF [22].

In the context of the PREDIMED (PREvención con DIeta MEDiterránea) trial, a large nutrition intervention trial, some multimetabolite lipid patterns or lipid network-clusters were previously reported to be prospectively associated with either major cardiovascular disease (coronary heart disease, stroke or cardiovascular death), heart failure, or type 2 diabetes [29, 30, 34, 37, 38], suggesting that lipidomics networks may also be related to AF risk. Moreover, the incidence of AF was significantly reduced in the PREDIMED arm using a Mediterranean diet (MedDiet) supplemented with extra-virgin olive oil (EVOO) [22], suggesting that modification of the dietary lipid content may mitigate AF risk, especially because an inverse dose–response effect was found between the actual percentage of caloric intake provided by EVOO and the subsequent risk of AF. However, comprehensive information on the association of lipidome profiles with future AF incidence is still scarce, and evidence on the joint association of interrelated lipid metabolites with new-onset AF has seldom been assessed [33]. We aimed to assess if participants’ baseline lipidome profile in the PREDIMED trial was associated with incident AF and whether the dietary intervention conducted in the PREDIMED trial could modify this association.

## Methods

The present case–control study was nested in the PREDIMED trial [10, 21]. Briefly, the PREDIMED trial was a multicenter randomized primary prevention trial designed to assess the effect of the MedDiet on CVD. Participants were recruited during 2003–2009. Participants were 7447 men (aged 55 to 80 years) and women (aged 60 to 80 years) without prior CVD but at high cardiovascular risk due to the presence of either type 2 diabetes or at least three of the following classical cardiovascular risk factors: current smoking, overweight/obesity, high LDL-cholesterol, low HDL-cholesterol, family history of early coronary heart disease, or hypertension. Participants were randomly allocated in a 1:1:1 ratio to a MedDiet supplemented with EVOO (MedDiet + EVOO), to a MedDiet supplemented with mixed tree nuts (MedDiet + nuts) or to a control group (advised to follow a low-fat diet and to reduce all types of fat). Participants received individual and group dietary educational sessions by a trained dietitian at the baseline visit and quarterly thereafter. Participants in the control group also received dietary education promoting a low-fat diet with the same intensity and frequency as the two MedDiet groups [21].

The trial was stopped because of early benefit based on endpoints documented through December 1, 2010, after a median intervention time and follow-up of 4.8 years.

**Fig. 1:**
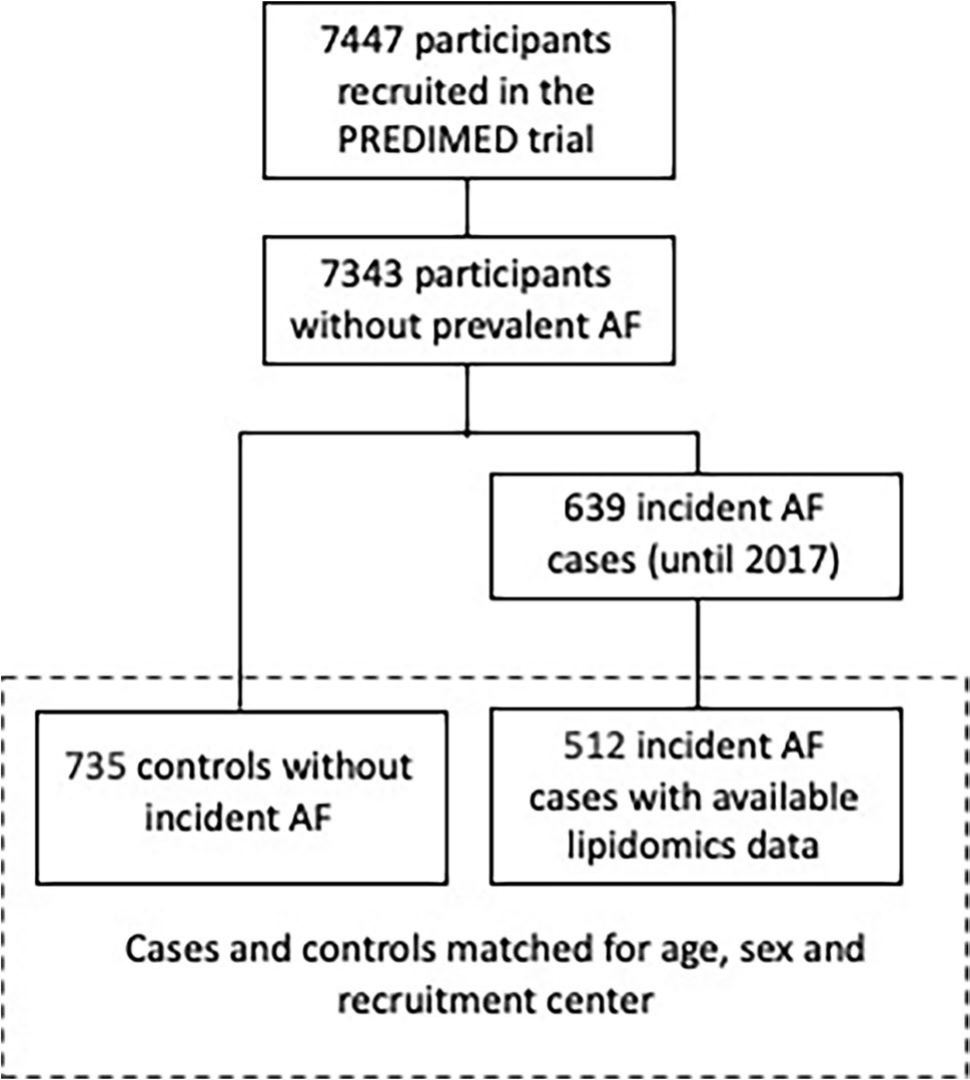
Flowchart of participants in the PREDIMED trial

Out of the 7447 participants in the PREDIMED trial, 7343 had no AF at study inception and 639 incident cases were registered (Fig. 1).

For the present study, we used a nested case–control design, where controls were matched by recruitment center, year of birth (± 5 years), and sex. We selected 1 to 3 matched controls per case, depending on availability of controls with the matched characteristics. Finally, we were able to include 512 incident cases of AF and 735 matched controls. We used the incidence density sampling (risk-set sampling) with replacement for selecting controls: controls were randomly selected from all eligible participants at risk at the time of the incident case occurrence, and selected controls could be selected again as a control for another index case and they could become later a case [28].

All participants completed a detailed battery of questionnaires at baseline which included information on sociodemographic characteristics and medical conditions, a validated Spanish translation of the Minnesota leisure time physical activity questionnaire [9] and a validated 14-item screener on their adherence to the traditional MedDiet [32].

The Institutional Review Board of the coordinating center approved the PREDIMED extended follow-up protocol (HCB/2019/0629), and of the Harvard TH Chan School of Public Health (00,002,642) and of the University of Navarra (2017.154_2012.104) approved the case–control subproject. All participants gave written informed consent.

At baseline, participants underwent a blood draw after an overnight fast (> 8 h) by trained technicians. Samples were processed by the study personnel according to the study protocol. EDTA plasma samples were coded and kept refrigerated until they were stored at − 80 °C in freezers.

For the present work, stored samples were shipped on dry ice to the Broad Institute for metabolomics analysis in 2018. Samples from cases-control participants were randomly distributed before being shipped to the Broad Institute in Boston, MA, for metabolomics assays.

### Metabolomic analyses of plasma

A detailed description on the determinations of plasma lipids can be found in the supplemental materials.

### Endpoint ascertainment

AF was a pre-specified secondary outcome in the PREDIMED trial as previously described elsewhere in detail [22] (see supplemental material).

### Statistical analysis

Missing values for the individual lipids in the PREDIMED trial were imputed with half of the minimum detected value. Baseline individual lipid values were normalized and scaled in multiples of 1 SD with Blom’s rank-based inverse normal transformation in the PREDIMED trial [3].

### Association between individual lipids and atrial fibrillation

We estimated the association for each individual lipid with a conditional logistic regression model adjusted for recruitment site, age, sex, smoking (3 categories), body mass index, type 2 diabetes, hypertension, family history of premature coronary heart disease, leisure time physical activity, educational level, statin use and intervention group. *p* Values were adjusted for multiple hypothesis testing with the procedure described by Benjamini and Yekutieli [2].

### Network and clustering

We used the conditional independence-based PC-algorithm for causal structure learning to generate the lipidomics network in the PREDIMED trial [15, 20]. This algorithm was applied to the same lipidomics data in another nested case–control study in the PREDIMED-trial on HF [38]. We retained edges with a partial correlation > 0.1 which were robust across the two case–control studies. We applied the walktrap algorithm in the igraph R-package (http://igraph.org/) to the robust lipidomics network to detect densely connected lipid clusters.

### Random forest-based evaluation of cluster importance

We selected the most important lipid clusters for AF prediction with a machine-learning-based selection in the PREDIMED trial. We prepared the lipidomics data based on deviations calculated from matching-strata-specific means to keep the matched design. We grew a random forest for AF-prediction based on the transformed lipids (500 trees, sampling rate of 2/3). The importance of lipid clusters for AF prediction was then evaluated in the out-of-bag sample. Hitherto, we assessed the random forest model’s predictive performance based on information on the full lipidomics data compared to the predictive performance based on lipidomics data with the joint permutation of lipid variables in each of the clusters. Clusters were ranked by the extent to which omitting the information in their variables hampered the predictive performance, with the largest increase in prediction error corresponding to the highest cluster importance.

### Determining atrial fibrillation-predictors within important clusters and definition of a lipid score

To select the accountable lipid pattern for high cluster importance, for each of the most important clusters, we mutually included all cluster-variables in a regression model. We ran a tenfold cross-validated elastic net regression to select the robust AF predictors within each of the clusters in the PREDIMED trial.

Finally, we estimated a weighted score combining all the metabolites described in the last step applying the leave-one-out cross-validation approach in conditional logistic regression models adjusted for age, sex, smoking habit, body-mass index, type 2 diabetes, hypertension, family history of coronary heart disease, leisure-time physical activity, education (3 levels), statin use, and intervention group (3 groups). We addressed the possible interaction between the weighted score and the intervention (3 groups) with the Wald test.

### Principal component analysis

We ran a principal component analysis (PCA) with the 216 lipid metabolites among the controls in the PREDIMED trial. Those factors with an eigenvalue higher than 2 were retained. The varimax rotation was applied. Sixteen uncorrelated factors were extracted. The extracted factors explained 83% of the total variance. Individual metabolites with absolute loadings > 0.40 were considered relevant components of the identified factors. Loadings obtained among controls were then applied to the cases. To analyze the association between the extracted factors and incident AF, factors were categorized into quartiles based on the distribution among controls. We also calculated the p for trend by estimating quartile-specific medians and treating the resulting variable as continuous. We addressed the possible effect modification by the intervention (3 groups) by testing interaction product-terms (Wald test).

Analyses were performed with Stata SE 15.1 (College Station, TX) and R 3.6.3.

### Data and code sharing

After submission of a brief proposal and statistical analysis plan to the corresponding author and upon approval from the Predimed Steering Committee and Institutional Review Boards, the data and the code for data analyses will be made available to them using an onsite secure access data enclave.

## Results

Baseline characteristics of AF cases and controls are displayed in Table 1. In both cases and controls, mean baseline age was 68.5 years and 49% of the participants were women. The average BMI was 30 kg/m^2^. Nearly half of the participants had type 2 diabetes, and more than 80% had hypertension at baseline.

**Table 1:** Baseline characteristics of cases and controls

	Cases of AF	Matched controls^*^	*p* value
*n*	512	735	
Age, years	68.3 (6.1)	68.7 (6.1)	0.025
Sex, % women	49.8	48.0	N/A
Body mass index, kg/m^2^	30.7 (3.8)	29.7 (3.7)	< 0.001
Type 2 diabetes, %	48.1	49.7	0.660
Hypertension, %	88.3	82.3	0.004
Dyslipidemia, %	64.8	68.4	0.088
Statin use, %	36.1	35.9	0.821
Family history of coronary heart disease, %	19.0	19.3	0.732
Smoking habit, %			
Never smoker	58.8	57.6	
Current smoker	14.3	12.9	
Former smoker	27.0	29.5	0.623
Educational level, %			
Primary school or less	76.2	79.5	
Secondary or high-school	17.2	14.4	
College or higher	6.6	6.1	0.217
Baseline adherence to the Mediterranean diet (0–14 score)	9.7 (2.0)	9.7 (1.9)	0.827
Leisure-time physical activity, METs-min/day	228 (217)	229 (217)	0.857
Intervention group, %			0.262
Control	36.9	35.7	
Mediterranean diet supplemented with extra-virgin olive oil	31.8	36.1	
Mediterranean diet supplemented with nuts	31.3	28.3	

*p* Values were obtained from conditional logistic regression models

*N/A* does not apply

^*^Controls were randomly selected from all participants at risk at the date of atrial fibrillation diagnoses (incidence density sampling) so that cases were potentially eligible as controls before developing atrial fibrillation, matched by recruitment center, year of birth (± 5 years), and sex

### Association between individual lipids and atrial fibrillation

The associations between each baseline individual lipid and incident AF are displayed in Supplemental Table 1. Forty-one lipids were nominally (*p* < 0.05) associated with incident AF, but none remained associated after multiple testing adjustment (all FDR > 0.05).

### Lipid networks and their association with atrial fibrillation

**Fig. 2:**
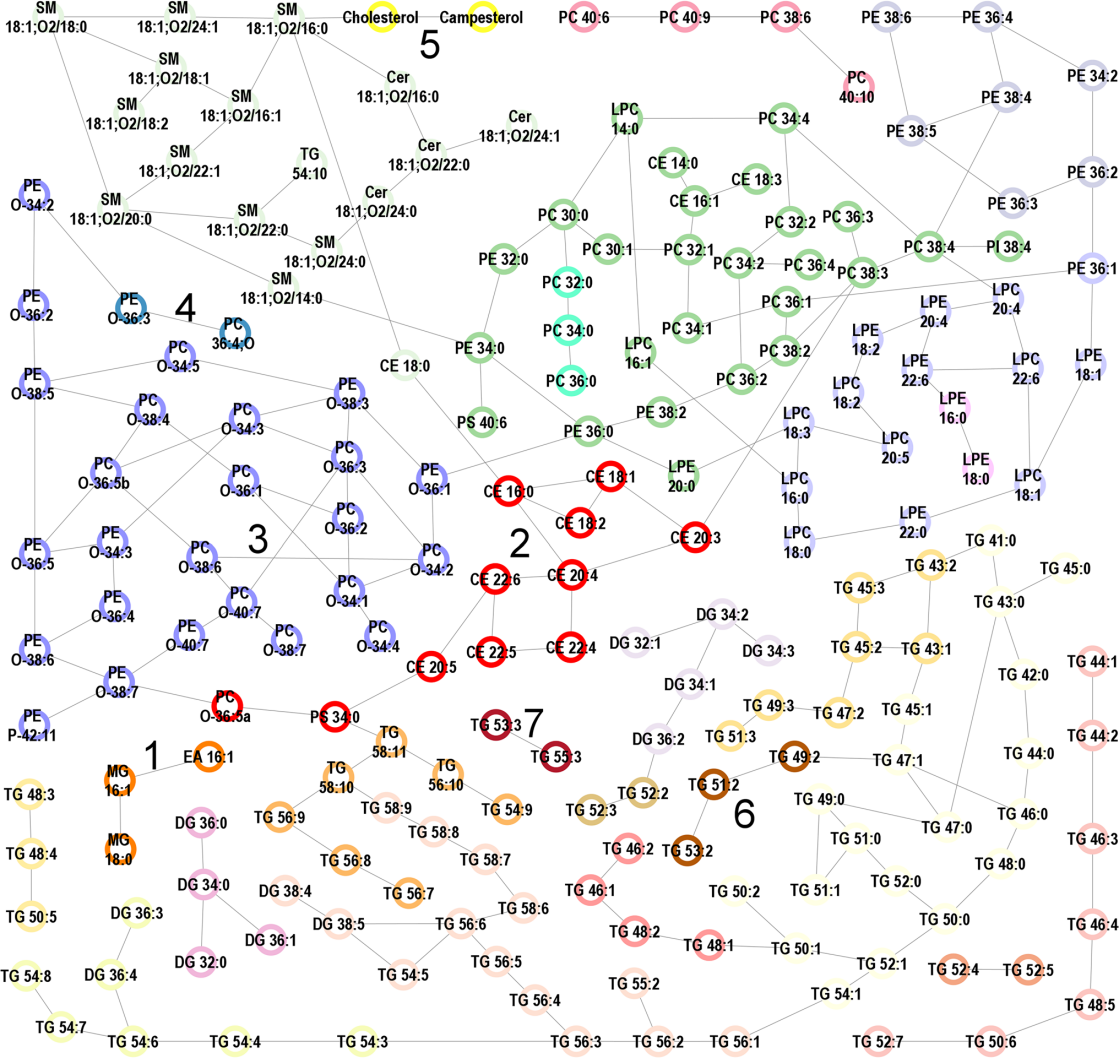
Lipid network and color-coded walktrap-clusters

Figure 2 shows the robust data-driven lipid network based on the conditional independence structure, which integrated 216 lipids. Generally, the data-derived clusters included lipids from the same or metabolically closely related lipid classes.

Table 2 shows the most important lipid clusters identified in the random-forest-based evaluation. They were ranked according to the extent to which omitting the information in their variables hampered the predictive performance. For example, omitting the information on MG 16:1, MG 18:0, and palmitoyl-EA (cluster 1-lipids) resulted in the largest increase in prediction error.

**Table 2:** Most important lipid clusters for AF-prediction identified in the random-forest based evaluation

Rank	Cluster lipids
1	MG 16:1, MG 18:0, palmitoyl-EA
2	CE 16:0, CE 18:1, CE 18:2, CE 20:4, CE 20:5, CE 22:5, CE 22:6, PS 34:0, PC P-36:4 or PC O-36:5, CE 20:3, CE 22:4
3	PC P-34:0 or PC O-34:1, PC P-34:1 or PC O-34:2, PE P-34:1 or PE O-34:2, PC P-34:2 or PC O-34:3, PE P-34:2 or PE O-34:3, PC P-34:3 or PC O-34:4, PC P-34:4 or PC O-34:5, PC P-36:0 or PC O-36:1, PE P-36:0 or PE O-36:1, PC P-36:1 or PC O-36:2, PE P-36:1 or PE O-36:2, PC P-36:2 or PC O-36:3, PE P-36:3 or PE O-36:4, PC P-36:4 or PC O-36:5, PE P-36:4 or PE O-36:5, PC P-38:3 or PC O-38:4, PC P-38:5 or PC O-38:6, PC P-38:6 or PC O-38:7, PE P-38:6 or PE O-38:7, PC P-40:6 or PC O-40:7, PE P-38:2 or PE O-38:3, PE P-38:4 or PE O-38:5, PE P-40:6 or PE O-40:7, PE P-38:5 or PE O-38:6, PE P-42:10 or PE O-42:11
4	PC 36:4;O, PE P-36:2 or PE O-36:3
5	Cholesterol, campesterol
6	TG 49:2, TG 51:2, TG 53:2
7	TG 53:3, TG 55:3

Lipid clusters were ranked according to the extent to which omitting the information in their variables hampered the predictive performance

We selected within-cluster AF predictive lipids for each important cluster with cross-validated elastic net regression (Supplemental Table 2). Then, we selected network-wide independent AF predictors by tenfold cross-validated elastic net regression with all selected within-cluster predictors as potential explanatory variables. Table 3 shows the network-wide, mutually independent AF predictors. The selected lipids included fourteen plasmalogens (8 with positive weights, 6 with inverse weights), as well as palmitoyl-EA and CE 16:0, which were positively weighted, and PC 36:4;O, cholesterol, and TG 53:2 and 53:3 which were inversely weighted.

**Table 3:** Mutually independent robust independent atrial fibrillation predictors across all selected network clusters and their median values in the leave-one-out cross-validation

Across-cluster selected lipids	Coefficient from the ENR	Median value from the LOOCV^*^
Palmitoyl-EA	0.095	0.090
CE 16:0	0.126	0.167
PC P-36:4 or PC O-36:5	− 0.025	− 0.012
PC P-34:1 or PC O-34:2	0.316	0.464
PE P-34:2 or PE O-34:3	0.380	0.527
PC P-34:4 or PC O-34:5	0.030	0.078
PC P-36:0 or PC O-36:1	0.108	0.165
PE P-36:0 or PE O-36:1	0.015	− 0.001
PC P-36:1 or PC O-36:2	− 0.232	− 0.306
PE P-36:3 or PE O-36:4	− 0.374	− 0.500
PC P-38:3 or PC O-38:4	0.094	0.097
PC P-38:5 or PC O-38:6	− 0.099	− 0.163
PE P-38:2 or PE O-38:3	− 0.216	− 0.271
PC 36:4;O	− 0.176	− 0.206
PE P-36:2 or PE O-36:3	0.157	0.207
Cholesterol	− 0.108	− 0.136
TG 53:3	− 0.016	0.019

*ENR* elastic net regression, *LOOCV* leave-one-out cross-validation

^*^Results from conditional logistic regression models adjusted for age, sex, smoking habit (3 categories), body mass index, prevalent hypertension, prevalent type-2 diabetes, family history of coronary heart disease, leisure-time physical activity (continuous), educational level (3 categories), and intervention group

Finally, we summarized the selected lipid predictors’ association with AF risk in a weighted sum score. The weights were generated in a leave-one-out cross-validation procedure based on the selected lipid predictors. The multivariable-adjusted OR for AF incidence per SD increase in the lipid score was 1.32 (95% CI 1.16 to 1.51; *p* < 0.001). There was no evidence of an effect modification by the dietary intervention of the PREDIMED trial (*p* value > 0.05).

### Results from the PCA and the association between the identified factors and atrial fibrillation

Out of the 16 extracted factors, 3 factors (factors 5, 11, and 15) showed associations (nominal *p* values < 0.05) with incident AF and the individual lipids with a factor loading > 0.40 in these factors are displayed in Supplemental Table 3. Supplemental Table 4 shows the ORs between the quartiles of each of the 3 factors identified in the PCA and AF. The strongest association was an inverse association observed for factor 5 (labeled as “low in cholesterol esthers and high in di- and triacylglycerols”): OR_5th vs. 1st quartile_ 0.64 (95% CI 0.45–0.90) (*p* for trend: 0.027). This factor was poor in rather saturated cholesterol esters and rich in diacylglycerols and triacylglycerols with a rather long chain and an intermediate degree of unsaturation.

None of these factors showed an interaction with the intervention group (*p* value > 0.05).

## Discussion

In this nested case–control within the PREDIMED trial, after adjusting for multiple testing, no single lipid was associated with AF incidence. However, a network-based multilipid combined score identified using a robust data-driven lipid network was associated with AF risk. In this combined score, several PC-plasmalogens and PE-plasmalogens were positively weighted whereas others were inversely weighted; in addition, palmitoyl-EA and were positively weighted, and cholesterol, PC 36:4;O, and TG 53:3 were negatively weighted.

Our results are consistent with results from the ARIC Study and the Framingham Heart Study [1, 17] in which no single lipid molecule was found to be prospectively associated with AF after adjustment for multiple testing. In the ARIC Study, only a secondary bile acid (glycocholenate sulfate), pseudouridine and acisoga were directly associated with AF onset and uridine was inversely associated with AF. Notwithstanding, SM 16:0 was directly associated with the risk of developing AF and this lipid was also found to be directly associated with AF risk in a previously published cohort [13]. In that study, ceramide with palmitic acid was also associated with AF risk but this association was not replicated in our study.

Despite the lack of associations between individual lipids and AF, we combined network analysis and machine learning to select a subset of lipids jointly associated with AF risk in PREDIMED. We summarized the joint association of these lipids with AF risk in a weighted sum score, using weights from a leave-one-out cross-validation procedure to avoid model overfitting. The estimated score included information on 17 lipids, out of which 7 were PC-plasmalogens and 5 were PE-plasmalogens. The short- and medium-chain and less unsaturated PC-plasmalogens with positive coefficients and medium-chain and more unsaturated PC-plasmalogens with negative coefficients. Similarly, shorter PE-plasmalogens showed positive coefficients and medium-chain PE-plasmalogens showed negative coefficients. These results are in line with the results of a case control study on AF, in which they observed in partial least square-discriminant analysis that a lower degree of unsaturation of LPC, LPE and PC levels was associated with a higher odds of AF [14]. Also, in the latter study, a relatively high degree of unsaturation was associated with a lower odds of AF. On the other hand, the other prospective study whose authors conducted a cluster analyses was the ADVANCE trial. They found that a cluster containing GM3(d18:1/24:1) and closest related lipid species was relevant to prediction of future AF. Unfortunately, we could not assess the consistency with their results since those lipids were not available in our platform [33].

Lipoproteins transport plasmalogens, and nearly one-third of the human heart’s phospholipids are plasmalogens. Plasmalogens could play a role in AF prevention through different pathways [8, 36]. Plasmalogens have antioxidant properties, since their free radical-scavenging chemical structure may decrease oxidative stress sensitivity [4, 24]. These properties support our findings’ biological plausibility—shorter and more saturated plasmalogens were positively associated with the multilipid score and inverse weights were observed for longer and more unsaturated plasmalogens—given that atrial myocardial inflammation and oxidative stress may play a crucial role in AF development [4, 8, 24, 36].

Plasmalogens have also been suggested to be PPARγ agonists [7], which may prevent cardiac fibrosis through different pathways, according to experimental models [19]. First, PPARγ can regulate inflammation through the NF-κB pathway reducing the risk of cardiac fibrosis and AF. In addition, PPARγ agonists could decrease the number of myofibroblasts, collagen I and brain natriuretic peptide (BNP) expression, matrix metalloproteinases-2 (MMP-2) activity, and protein level of connective tissue growth factor (CTGF). PPARγ agonists could also attenuate atrial structural remodeling, regulate cardiac telomere biology, alleviate MMP-9 activity, and reduce vascular endothelial dysfunction by inhibition of cardiovascular NADPH oxidase, among others. Additionally, plasmalogens may contribute to the stability of lipid raft microdomains and improved vesicular function [27, 35] and they may be required for cholesterol transport from the plasma membrane to the endoplasmatic reticulum [23]. In the identified multilipid score, some plasmalogens were associated with a higher risk of AF whereas others showed an inverse association. In our study, shorter and more saturated PC-plasmalogens showed positive coefficients and medium-chain and more unsaturated PC-plasmalogens showed negative coefficients. Thus, not only plasmalogens as a whole but also the fatty acid composition of plasmalogens may play an etiological role in AF development as it is the case for other lipid species [14]. Nevertheless, more evidence is needed for the potential association between plasmalogens as a whole and specific plasmalogens and atrial fibrillation.

In the multilipid score, palmitoyl-EA showed a positive association with incident AF. This observation does not easily align with experimental evidence, suggesting possible anti-inflammatory properties of palmitoyl-EA [26]. A possible explanation may be compensatory upregulation of anti-inflammatory factors, which has been observed to precede chronic disease onset. Nevertheless, we have no clear explanation for this finding.

In the multilipid score, free cholesterol was inversely associated with incident AF. Evidence on the association between free cholesterol and incident AF is scarce. Nevertheless, in a recent meta-analysis, total cholesterol was inversely associated with this condition [11].

In PREDIMED, participants allocated to the MedDiet + EVOO group showed a 38% lower risk of AF than those allocated to the control group, whereas participants allocated to the MedDiet + nuts group showed no reduction in the risk of AF [22]. We observed no effect modification by the interventions for the association between the multilipid score and AF. This result suggests that the intervention did not counterbalance the possible deleterious association between the multilipid score and incident AF and that the underlying mechanisms in the association between the intervention and AF may be different from the lipid profile modification, possibly suggesting that some nonlipid constituents of EVOO, such as phenolic compounds with potent antioxidant and anti-inflammatory properties, may be important for disease prevention [31].

We acknowledge that our study had some limitations. First, we could not distinguish between different isomers of a given lipid formula in our primary analyses, and different isomeric forms of some lipids may show a differential association with AF. Second, participants in the PREDIMED trial were at high cardiovascular risk and mainly Caucasians, limiting the generalizability of our results to other populations. Third, despite the multivariable adjustment, residual confounding cannot be ruled out.

Despite the abovementioned limitations, our study also shows some strengths. We used different dimension-reduction methods to disentangle potential lipidome patterns associated with the risk of developing AF. To our knowledge, this is one of the few prospective studies on AF risk that have addressed the assessment of interplays between different lipids and has not only considered individual lipids in isolation [33]. Also, we have applied machine learning methods to combine different metabolites to avoid overfitting. This methodological advance increases the robustness and reliability of our results.

In conclusion, no individual lipids were prospectively associated with the risk of AF after penalization for multiple comparisons. However, a weighted multilipid score, primarily made up of plasmalogens, was associated with AF risk. Further studies are needed to examine our observations’ generalizability to other populations and elucidate the underlying biological mechanisms.

## Supplementary Information

Below is the link to the electronic supplementary material.

Supplementary file1 (DOCX 41.1 KB)
