# Supplementary material for: Plasma lipidome and risk of atrial fibrillation: results from the PREDIMED trial
Source: J Physiol Biochem. 2023 Apr 1;79(2):355–64. doi: 10.1007/s13105-023-00958-0 (PMC10300169; doi:10.1007/s13105-023-00958-0)
Supplement: Supplementary file 1 — Supplementary file1 (DOCX 41.1 KB) [file 13105_2023_958_MOESM1_ESM.docx]

*Metabolomic analyses of plasma*

Plasma polar and nonpolar lipids were profiled using a Nexera X2 U-HPLC system (Shimadzu Scientific Instruments; Marlborough, MA) coupled to an Exactive Plus orbitrap mass spectrometer (Thermo Fisher Scientific; Waltham, MA). Lipids were extracted from plasma (10 µL) using 190 µL of isopropanol containing 1,2-didodecanoyl-sn-glycero-3-phosphocholine as an internal standard (Avanti Polar Lipids; Alabaster, AL). After centrifugation (10 min, 9,000 x g, ambient temperature), supernatants (10 µL) were injected directly onto a 100 x 2.1 mm ACQUITY BEH C8 column (1.7 µm; Waters; Milford, MA). The column was eluted at a flow rate of 450 µL/min isocratically for 1 minute at 80% mobile phase A (95:5:0.1 vol/vol/vol 10 mM ammonium acetate/methanol/acetic acid), followed by a linear gradient to 80% mobile-phase B (99.9:0.1 vol/vol methanol/acetic acid) over 2 minutes, a linear gradient to 100% mobile phase B over 7 minutes, and then 3 minutes at 100% mobile-phase B. MS analyses were carried out using electrospray ionization in the positive ion mode using full scan analysis over m/z 200-1100 at 70,000 resolution and 3 Hz data acquisition rate. Additional MS settings were: ion spray voltage, 3.0 kV; capillary temperature, 300°C; probe heater temperature, 300 °C; sheath gas, 50; auxiliary gas, 15; and S-lens RF level 60. To enable quality control analyses (QC) and drift correction, a pooled plasma QC sample was created by pooling a small aliquot of every study sample while they were aliquoted for these analyses. Pairs of pooled plasma QC samples were inserted into the analytical queue at intervals of 20 study samples. One pooled QC sample from each pair was used to correct for analytical drift using a nearest neighbor approach and the second pooled plasma sample served as a passive QC sample to evaluate analytical reproducibility of every lipid. Internal standard signals were evaluated while the samples were being analyzed to identify outliers for re-analysis and data from pooled plasma samples were manually inspected every day while the analytical queue was running to assure consistent analytical performance [1]. Raw data were processed using Progenesis QI software (NonLinear Dynamics) for feature alignment, nontargeted signal detection, and signal integration. Targeted processing of a subset of lipids was conducted using TraceFinder software (version 3.3, Thermo Fisher Scientific; Waltham, MA). Lipids are denoted by headgroup and total acyl carbon content and total acyl double bond content.

Since reference standards were not available to confirm identities of all measured lipid species, representative lipids from each lipid class were used to characterize retention time (RT) and mass to charge ratio (m/z) ratio patterns. Lipid identities are reported at the level of lipid class, total acyl carbon content, and total double bond content since the LC-MS method does not discretely resolve all isomeric lipids from one another. Measured mass-to-charge ratios (m/z) and measured retention times (RT) can be found in supplemental table 5.

*Endpoint ascertainment*

AF was initially identified from an annual review of all outpatient and inpatient medical records of each participant and yearly ECGs performed during follow-up examinations in the healthcare centers. If a medical diagnosis of AF was mentioned anywhere in the annually reviewed medical records of the participant or AF was present in any ECG, all relevant documentation was submitted to the Clinical End-point Committee. Even though AF was not a primary endpoint in the trial, the Clinical End-point Committee reviewed all medical charts and ECGs from potential AF cases and made a final decision adjudicating he presence or absence of atrial fibrillation. A confirmed diagnosis of atrial fibrillation was adjudicated only if both AF was present in an ECG tracing and an explicit medical diagnosis of AF was made by a physician. Atrial fibrillation events associated with MI or cardiac surgery (usually transient) were not included. For the present analysis, all AF cases up to December 31^st^ 2017 –except for one recruitment center with available follow-up until December 31^st^ 2014– with available plasma samples and lipidomics measurements were included.

**References**

1. Chaffin MD, Cao L, Deik AA, Clish CB, et al. MetProc: Separating Measurement Artifacts from True Metabolites in an Untargeted Metabolomics Experiment. J Proteome Res. 2019;18(3):1446-1450.

Supplemental table 1. Association between individual lipids and atrial fibrillation

| **Metabolite** | **OR** | **95% CI** | **p-value** |
| --- | --- | --- | --- |
| TG 52:2 | 0.82 | (0.72,0.93) | 0.002 |
| CE 16:0 | 1.22 | (1.08,1.39) | 0.002 |
| DG 36:2 | 0.83 | (0.73,0.94) | 0.003 |
| SM 18:1;O2/14:0 | 1.21 | (1.07,1.37) | 0.003 |
| TG 54:2 | 0.84 | (0.74,0.95) | 0.005 |
| TG 56:2 | 0.84 | (0.74,0.95) | 0.006 |
| TG 55:2 | 0.85 | (0.75,0.96) | 0.008 |
| DG 34:1 | 0.85 | (0.75,0.96) | 0.010 |
| TG 52:3 | 0.85 | (0.75,0.96) | 0.010 |
| TG 56:3 | 0.85 | (0.75,0.96) | 0.010 |
| PC P-36:0 or PC O-36:1 | 1.18 | (1.04,1.34) | 0.011 |
| TG 52:6 | 0.85 | (0.75,0.96) | 0.011 |
| DG 36:1 | 0.84 | (0.74,0.96) | 0.011 |
| PE 36:1 | 0.85 | (0.75,0.97) | 0.013 |
| CE 18:1 | 1.17 | (1.03,1.34) | 0.014 |
| TG 50:2 | 0.86 | (0.77,0.97) | 0.018 |
| TG 50:5 | 0.86 | (0.76,0.97) | 0.018 |
| PC P-34:1 or PC O-34:2 | 1.17 | (1.03,1.34) | 0.019 |
| TG 52:7 | 0.86 | (0.75,0.98) | 0.019 |
| DG 34:3 | 0.86 | (0.76,0.98) | 0.021 |
| SM 18:1;O2/16:0 | 1.17 | (1.02,1.33) | 0.022 |
| PC38:3 | 0.86 | (0.76,0.98) | 0.022 |
| TG 50:1 | 0.87 | (0.77,0.98) | 0.024 |
| TG 54:8 | 0.86 | (0.76,0.98) | 0.025 |
| PC40:6 | 0.87 | (0.76,0.98) | 0.027 |
| DG 34:2 | 0.87 | (0.77,0.99) | 0.031 |
| TG 50:3 | 0.88 | (0.78,0.99) | 0.032 |
| TG 53:2 | 0.88 | (0.78,0.99) | 0.032 |
| TG 54:7 | 0.87 | (0.77,0.99) | 0.036 |
| DG 32:1 | 0.88 | (0.77,0.99) | 0.038 |
| PE 38:6 | 0.87 | (0.77,0.99) | 0.038 |
| CE 20:4 | 1.14 | (1.01,1.29) | 0.039 |
| CE 18:0 | 1.14 | (1.01,1.3) | 0.039 |
| TG 51:3 | 0.88 | (0.78,0.99) | 0.039 |
| CE 18:2 | 1.14 | (1.01,1.29) | 0.041 |
| TG 41:0 | 0.87 | (0.76,0.99) | 0.042 |
| DG 38:5 | 0.87 | (0.76,1) | 0.042 |
| TG 50:4 | 0.88 | (0.78,1) | 0.043 |
| DG 38:4 | 0.88 | (0.77,1) | 0.045 |
| TG 47:0 | 0.88 | (0.78,1) | 0.046 |
| TG 43:1 | 0.88 | (0.78,1) | 0.047 |
| TG 56:1 | 0.88 | (0.78,1) | 0.051 |
| Cer 18:1;O2/24:1 | 0.88 | (0.78,1) | 0.051 |
| TG 50:6 | 0.88 | (0.77,1) | 0.053 |
| DG 36:3 | 0.88 | (0.78,1) | 0.055 |
| TG 54:3 | 0.89 | (0.79,1) | 0.056 |
| TG 54:5 | 0.88 | (0.77,1) | 0.057 |
| TG 49:3 | 0.89 | (0.79,1) | 0.058 |
| TG 52:1 | 0.89 | (0.79,1.01) | 0.061 |
| TG 48:3 | 0.89 | (0.79,1.01) | 0.061 |
| PE 36:2 | 0.88 | (0.78,1.01) | 0.063 |
| PE 38:5 | 0.88 | (0.77,1.01) | 0.069 |
| TG 56:4 | 0.89 | (0.78,1.01) | 0.073 |
| TG 42:0 | 0.89 | (0.79,1.01) | 0.073 |
| TG 56:7 | 0.89 | (0.79,1.01) | 0.078 |
| PE 38:4 | 0.89 | (0.77,1.01) | 0.078 |
| TG 48:5 | 0.89 | (0.78,1.01) | 0.080 |
| TG 48:1 | 0.9 | (0.8,1.01) | 0.080 |
| TG 49:0 | 0.9 | (0.8,1.01) | 0.084 |
| TG 51:2 | 0.9 | (0.8,1.02) | 0.088 |
| PE 40:6 | 0.89 | (0.78,1.02) | 0.089 |
| TG 48:4 | 0.9 | (0.79,1.02) | 0.091 |
| SM 18:1;O2/16:1 | 1.14 | (0.98,1.33) | 0.092 |
| PC P-38:3 or PC O-38:4 | 1.11 | (0.98,1.26) | 0.093 |
| CE 22:5 | 1.11 | (0.98,1.26) | 0.094 |
| PE P-36:2 or PE O-36:3 | 1.12 | (0.98,1.28) | 0.097 |
| TG 47:2 | 0.9 | (0.8,1.02) | 0.097 |
| PE P-36:0 or PE O-36:1 | 1.11 | (0.98,1.25) | 0.098 |
| TG 52:5 | 0.9 | (0.8,1.02) | 0.100 |
| TG 53:3 | 0.9 | (0.8,1.02) | 0.102 |
| TG 44:0 | 0.9 | (0.8,1.02) | 0.107 |
| PC40:10 | 0.9 | (0.79,1.02) | 0.108 |
| TG 54:9 | 0.9 | (0.79,1.02) | 0.109 |
| DG 30:0 | 0.91 | (0.8,1.02) | 0.110 |
| TG 51:0 | 0.91 | (0.81,1.02) | 0.113 |
| TG 54:1 | 0.91 | (0.8,1.02) | 0.117 |
| TG 45:1 | 0.91 | (0.8,1.03) | 0.118 |
| TG 46:3 | 0.91 | (0.8,1.03) | 0.122 |
| TG 48:2 | 0.91 | (0.81,1.03) | 0.123 |
| TG 43:2 | 0.91 | (0.8,1.03) | 0.127 |
| CE 14:0 | 1.1 | (0.97,1.24) | 0.127 |
| TG 43:0 | 0.91 | (0.8,1.03) | 0.130 |
| DG 32:2 | 0.91 | (0.81,1.03) | 0.130 |
| TG 45:2 | 0.91 | (0.81,1.03) | 0.132 |
| TG 56:9 | 0.91 | (0.8,1.03) | 0.135 |
| PC P-34:0 or PC O-34:1 | 1.1 | (0.97,1.25) | 0.142 |
| PC P-36:2 or PC O-36:3 | 1.1 | (0.97,1.24) | 0.145 |
| TG 46:4 | 0.91 | (0.8,1.03) | 0.145 |
| TG 46:2 | 0.91 | (0.81,1.03) | 0.151 |
| TG 52:4 | 0.91 | (0.81,1.03) | 0.151 |
| PE 34:2 | 0.91 | (0.81,1.03) | 0.152 |
| TG 46:0 | 0.92 | (0.81,1.03) | 0.155 |
| TG 56:6 | 0.91 | (0.79,1.04) | 0.157 |
| PI 38:4 | 0.92 | (0.81,1.04) | 0.160 |
| CE 22:4 | 1.09 | (0.97,1.23) | 0.161 |
| TG 56:10 | 0.91 | (0.8,1.04) | 0.163 |
| PC38:2 | 0.92 | (0.81,1.04) | 0.166 |
| TG 45:3 | 0.92 | (0.81,1.04) | 0.169 |
| TG 58:7 | 0.91 | (0.8,1.04) | 0.172 |
| Palmitoyl-EA | 1.1 | (0.96,1.26) | 0.180 |
| TG 47:1 | 0.92 | (0.82,1.04) | 0.181 |
| PE 36:4 | 0.92 | (0.81,1.04) | 0.183 |
| PS 34:0 | 0.92 | (0.82,1.04) | 0.185 |
| LPC22:6 | 0.92 | (0.82,1.04) | 0.186 |
| PC38:6 | 0.92 | (0.81,1.04) | 0.187 |
| TG 48:0 | 0.92 | (0.82,1.04) | 0.190 |
| LPE18:1 | 0.92 | (0.81,1.04) | 0.192 |
| TG 54:4 | 0.92 | (0.82,1.04) | 0.194 |
| PC40:9 | 0.92 | (0.81,1.04) | 0.198 |
| PC 36:4;O | 0.9 | (0.76,1.06) | 0.199 |
| TG 46:1 | 0.92 | (0.82,1.04) | 0.202 |
| TG 44:2 | 0.92 | (0.82,1.04) | 0.204 |
| TG 44:1 | 0.92 | (0.82,1.04) | 0.208 |
| LPE22:6 | 0.92 | (0.82,1.05) | 0.209 |
| TG 49:1 | 0.93 | (0.82,1.04) | 0.210 |
| TG 52:0 | 0.92 | (0.82,1.05) | 0.214 |
| PC P-36:4a or PC O-36:5a | 0.93 | (0.82,1.05) | 0.220 |
| PE P-34:1 or PE O-34:2 | 1.08 | (0.95,1.23) | 0.226 |
| TG 49:2 | 0.93 | (0.83,1.05) | 0.227 |
| DG 36:4 | 0.93 | (0.82,1.05) | 0.229 |
| TG 55:3 | 0.93 | (0.82,1.05) | 0.244 |
| PE 36:3 | 0.93 | (0.82,1.05) | 0.251 |
| MG 16:1 | 1.09 | (0.94,1.27) | 0.255 |
| PC32:0 | 1.08 | (0.95,1.22) | 0.262 |
| PC P-34:2 or PC O-34:3 | 1.07 | (0.95,1.2) | 0.270 |
| SM 18:1;O2/22:1 | 1.08 | (0.94,1.24) | 0.273 |
| TG 56:8 | 0.93 | (0.82,1.06) | 0.278 |
| PC36:1 | 0.94 | (0.83,1.06) | 0.294 |
| TG 51:1 | 0.94 | (0.84,1.06) | 0.299 |
| PE P-34:2 or PE O-34:3 | 1.07 | (0.94,1.2) | 0.300 |
| TG 50:0 | 0.94 | (0.83,1.06) | 0.302 |
| PE P-36:1 or PE O-36:2 | 1.07 | (0.94,1.21) | 0.320 |
| Coenzyme Q10 | 0.94 | (0.82,1.07) | 0.324 |
| TG 56:5 | 0.94 | (0.83,1.07) | 0.341 |
| TG 58:11 | 0.94 | (0.82,1.07) | 0.342 |
| PC34:3 | 0.94 | (0.83,1.07) | 0.346 |
| PC P-36:1 or PC O-36:2 | 1.06 | (0.93,1.2) | 0.363 |
| PC38:4 | 0.94 | (0.82,1.07) | 0.368 |
| PC32:1 | 0.95 | (0.84,1.07) | 0.371 |
| TG 58:8 | 0.94 | (0.83,1.07) | 0.371 |
| CE 20:3 | 1.06 | (0.94,1.19) | 0.374 |
| PE 34:0 | 1.06 | (0.93,1.2) | 0.380 |
| TG 58:6 | 0.94 | (0.83,1.08) | 0.384 |
| PC P-36:4b or PC O-36:5b | 1.05 | (0.94,1.18) | 0.388 |
| TG 60:12 | 0.95 | (0.83,1.08) | 0.397 |
| PC34:1 | 0.95 | (0.84,1.07) | 0.399 |
| PC36:3 | 0.95 | (0.84,1.07) | 0.402 |
| PE 36:0 | 1.06 | (0.93,1.2) | 0.409 |
| Piperine | 1.06 | (0.92,1.22) | 0.428 |
| PC P-34:4 or PC O-34:5 | 1.05 | (0.93,1.19) | 0.430 |
| PC34:0 | 1.05 | (0.93,1.19) | 0.435 |
| LPE18:0 | 0.95 | (0.84,1.08) | 0.458 |
| PC36:2 | 0.95 | (0.84,1.08) | 0.459 |
| LPC16:1 | 0.95 | (0.84,1.08) | 0.459 |
| PC P-34:3 or PC O-34:4 | 1.05 | (0.92,1.19) | 0.474 |
| PC P-40:6 or PC O-40:7 | 1.05 | (0.92,1.2) | 0.481 |
| DG 36:0 | 0.95 | (0.83,1.09) | 0.491 |
| SM 18:1;O2/18:2 | 1.05 | (0.91,1.2) | 0.509 |
| CE 22:6 | 1.04 | (0.92,1.17) | 0.511 |
| TG 54:6 | 0.96 | (0.85,1.08) | 0.522 |
| Sphingosine | 0.96 | (0.84,1.1) | 0.526 |
| CE 18:3 | 1.04 | (0.92,1.17) | 0.532 |
| SM 18:1;O2/20:0 | 1.04 | (0.92,1.18) | 0.540 |
| SM 18:1;O2/18:0 | 1.04 | (0.91,1.19) | 0.550 |
| Cer 18:1;O2/24:0 | 0.96 | (0.85,1.09) | 0.553 |
| DG 34:0 | 0.96 | (0.83,1.11) | 0.567 |
| PE P-38:4 or PE O-38:5 | 1.04 | (0.92,1.17) | 0.571 |
| PE P-42:10 or PE O-42:11 | 0.96 | (0.84,1.1) | 0.584 |
| Cer 18:1;O2/22:0 | 0.97 | (0.85,1.1) | 0.603 |
| Cer 18:1;O2/16:0 | 1.03 | (0.91,1.17) | 0.607 |
| TG 45:0 | 0.97 | (0.86,1.1) | 0.626 |
| LPE20:4 | 1.03 | (0.91,1.16) | 0.632 |
| TG 54:10 | 0.97 | (0.86,1.1) | 0.646 |
| PE P-38:6 or PE O-38:7 | 0.97 | (0.85,1.11) | 0.701 |
| TG 58:10 | 0.98 | (0.86,1.11) | 0.713 |
| LPE18:2 | 1.02 | (0.9,1.16) | 0.714 |
| TG 58:9 | 0.98 | (0.87,1.11) | 0.725 |
| PC34:2 | 1.02 | (0.9,1.16) | 0.729 |
| PE P-40:6 or PE O-40:7 | 1.02 | (0.89,1.17) | 0.734 |
| PC32:2 | 1.02 | (0.9,1.16) | 0.740 |
| PC30:1 | 0.98 | (0.87,1.1) | 0.749 |
| LPC16:0 | 1.02 | (0.9,1.16) | 0.754 |
| PE 32:0 | 1.02 | (0.9,1.16) | 0.758 |
| PC36:4 | 1.02 | (0.9,1.16) | 0.762 |
| LPE16:0 | 0.98 | (0.87,1.11) | 0.775 |
| SM 18:1;O2/18:1 | 1.02 | (0.88,1.18) | 0.791 |
| DG 32:0 | 0.98 | (0.84,1.14) | 0.800 |
| PE P-38:2 or PE O-38:3 | 1.02 | (0.9,1.15) | 0.801 |
| LPC14:0 | 1.01 | (0.9,1.15) | 0.829 |
| PC P-38:5 or PC O-38:6 | 0.99 | (0.87,1.12) | 0.833 |
| PE P-36:4 or PE O-36:5 | 1.01 | (0.9,1.14) | 0.840 |
| MG 18:0 | 1.02 | (0.87,1.19) | 0.841 |
| LPC18:1 | 0.99 | (0.87,1.12) | 0.846 |
| Cholesterol | 0.99 | (0.87,1.13) | 0.850 |
| PE P-38:5 or PE O-38:6 | 1.01 | (0.89,1.14) | 0.878 |
| SM 18:1;O2/24:1 | 1.01 | (0.89,1.15) | 0.884 |
| LPC18:0 | 1.01 | (0.89,1.15) | 0.884 |
| Oleoyl ethanolamine | 1.01 | (0.89,1.14) | 0.893 |
| PC36:0 | 1.01 | (0.89,1.15) | 0.898 |
| LPC20:5 | 0.99 | (0.88,1.12) | 0.902 |
| PE 38:2 | 1.01 | (0.89,1.14) | 0.909 |
| LPE22:0 | 1.01 | (0.88,1.15) | 0.918 |
| LPE20:0 | 1.01 | (0.89,1.15) | 0.918 |
| PE P-36:3 or PE O-36:4 | 0.99 | (0.88,1.12) | 0.922 |
| PC34:4 | 0.99 | (0.88,1.13) | 0.924 |
| LPC18:2 | 1.01 | (0.89,1.14) | 0.925 |
| PS 40:6 | 0.99 | (0.87,1.13) | 0.932 |
| LPC18:3 | 1 | (0.88,1.15) | 0.941 |
| SM 18:1;O2/24:0 | 1 | (0.89,1.14) | 0.948 |
| PC P-38:6 or PC O-38:7 | 1 | (0.89,1.14) | 0.956 |
| SM 18:1;O2/22:0 | 1 | (0.88,1.13) | 0.975 |
| PC30:0 | 1 | (0.89,1.12) | 0.976 |
| Campesterol | 1 | (0.87,1.15) | 0.994 |
| LPC20:4 | 1 | (0.88,1.13) | 0.994 |
| CE 20:5 | 1 | (0.89,1.13) | 0.994 |
| CE 16:1 | 1 | (0.89,1.13) | 0.999 |

Results from conditional logistic regression models adjusted for age, sex, smoking habit (3 categories), body-mass index, prevalent hypertension, prevalent type 2 diabetes, family history of coronary heart disease, leisure-time physical activity (continuous), educational level (3 categories), statin use and intervention group

Supplemental table 2: Robust atrial fibrillation-predictors within the selected network clusters

| **cluster** | **within-cluster selected lipids** | **coefficient from ENR** |
| --- | --- | --- |
| 1 | Palmitoyl-EA | 0.014 |
|  |  |  |
| 2 | CE 16:0 | 0.117 |
| 2 | PC P-36:4 or PC O-36:5 | -0.059 |
|  |  |  |
| 3 | PC P-34:1 or PC O-34:2 | 0.249 |
| 3 | PE P-34:2 or PE O-34:3 | 0.304 |
| 3 | PC P-34:4 or PC O-34:5 | 0.030 |
| 3 | PC P-36:0 or PC O-36:1 | 0.076 |
| 3 | PE P-36:0 or PE O-36:1 | 0.038 |
| 3 | PC P-36:1 or PC O-36:2 | -0.139 |
| 3 | PE P-36:3 or PE O-36:4 | -0.233 |
| 3 | PC P-36:4 or PC O-36:5 | 0.024 |
| 3 | PC P-38:3 or PC O-38:4 | 0.056 |
| 3 | PC P-38:5 or PC O-38:6 | -0.130 |
| 3 | PE P-38:2 or PE O-38:3 | -0.150 |
| 3 | PE P-42:10 or PE O-42:11 | -0.015 |
|  |  |  |
| 4 | PC 36:4;O | -0.136 |
| 4 | PE P-36:2 or PE O-36:3 | 0.082 |
|  |  |  |
| 5 | Cholesterol | -0.005 |
|  |  |  |
| 6 | TG 53:2 | -0.080 |
|  |  |  |
| 7 | TG 53:3 | -0.090 |

ENR: elastic net regression

Supplemental table 3. Individual lipids with a factor lading >0.40 in the factors that were associated to the risk of atrial fibrillation.

|  | Lipids with a negative factor loading | Lipids with a positive factor loading |
| --- | --- | --- |
| Factor 5 | CE 16:0; 18:2; 18:1; 18:0; 20:3; and 22:4 | LPE 18:1  PC 34:1; and 36:1  PE 36:2; and 36:1  DG 34:1; 36:3; 36:2; 36:1; and 38:4  TG 50:2; 50:1; 52:3; 52:2; 52:1; 53:2; 54:4; 54:3; 54:2; 54:1; 55:3; 55:2; 56:4; 56:3; 56:2; and 56:1 |
| Factor 11 |  | PC 34:3; 36:3; 38:3; and 38:2  CE 18:3 |
| Factor 15 |  | Palmitoyl-EA  MG 16:1; and 18:0  DG 36:0 |

Supplemental table 4. Odds ratios (95% CI) for the association between factors identified in the principal component analysis and incident atrial fibrillation in the PREDIMED trial

|  | Quartile 1 | Quartile 2 | Quartile 3 | Quartile 4 | p for trend |
| --- | --- | --- | --- | --- | --- |
| Factor 5 | 1 (ref) | 0.66 (0.48 to 0.91) | 0.83 (0.60 to 1.15) | 0.63 (0.45 to 0.89) | 0.025 |
| Factor 11 | 1 (ref) | 1.45 (1.03 to 2.04) | 1.11 (0.79 to 1.58) | 1.03 (0.72 to 1.47) | 0.782 |
| Factor 15 | 1 (ref) | 1.51 (1.03 to 2.20) | 1.31 (0.87 to 1.98) | 1.29 (0.83 to 2.01) | 0.389 |

Results from conditional logistic regression models adjusted for age, sex, smoking habit (3 categories), body-mass index, prevalent hypertension, prevalent type 2 diabetes, family history of coronary heart disease, leisure-time physical activity (continuous), educational level (3 categories), statin use and intervention group.

Supplemental table 5. Measured mass-to-charge ratios (m/z) and measured retention times (RT) for the different lipids

| **Lipid** | **M/Z** | **RT** |
| --- | --- | --- |
| PC 12:0/12:0 [iSTD] | 622.4450 | 6.54 |
| LPC 14:0 | 468.3095 | 4.37 |
| LPC 16:1 | 494.3251 | 4.53 |
| LPC 16:0 | 496.3403 | 4.88 |
| LPC 18:3 | 518.3226 | 4.75 |
| LPC 18:2 | 520.3407 | 4.71 |
| LPC 18:1 | 522.3564 | 5.05 |
| LPC 18:0 | 524.3721 | 5.44 |
| LPC 20:5 | 542.3228 | 4.60 |
| LPC 20:4 | 544.3408 | 4.74 |
| LPC 22:6 | 568.3408 | 4.71 |
| LPE 16:0 | 454.2935 | 4.90 |
| LPE 18:2 | 478.2936 | 4.73 |
| LPE 18:1 | 480.3094 | 5.07 |
| LPE 18:0 | 482.3253 | 5.46 |
| LPE 20:4 | 502.2934 | 4.75 |
| LPE 20:0 | 510.3565 | 5.15 |
| LPE 22:6 | 526.2932 | 4.73 |
| LPE 22:0 | 538.3876 | 5.76 |
| PC 30:1 | 704.5234 | 7.86 |
| PC 30:0 | 706.5389 | 8.19 |
| PC 32:2 | 730.5388 | 8.01 |
| PC 32:1 | 732.5546 | 8.35 |
| PC 32:0 | 734.5702 | 8.67 |
| PC 34:4 | 754.5389 | 8.04 |
| PC 34:3 | 756.5538 | 8.40 |
| PC 34:2 | 758.5703 | 8.50 |
| PC 34:1 | 760.5860 | 8.81 |
| PC 34:0 | 762.6014 | 9.11 |
| PC 36:4 | 782.5694 | 8.53 |
| PC 36:3 | 784.5857 | 8.67 |
| PC 36:2 | 786.6017 | 8.97 |
| PC 36:1 | 788.6174 | 9.25 |
| PC 36:0 | 790.6320 | 9.70 |
| PC 38:6 | 806.5700 | 8.46 |
| PC 38:4 | 810.6005 | 8.99 |
| PC 38:3 | 812.6170 | 9.12 |
| PC 38:2 | 814.6330 | 9.34 |
| PC 40:10 | 826.5370 | 8.16 |
| PC 40:9 | 828.5515 | 8.46 |
| PC 40:6 | 834.6010 | 8.92 |
| PC 36:4;O | 798.5654 | 7.40 |
| PC P-34:4 or PC O-34:5 | 738.5444 | 9.05 |
| PC P-34:3 or PC O-34:4 | 740.5567 | 8.73 |
| PC P-34:2 or PC O-34:3 | 742.5755 | 8.80 |
| PC P-34:1 or PC O-34:2 | 744.5910 | 9.11 |
| PC P-34:0 or PC O-34:1 | 746.6064 | 9.10 |
| PC P-36:4a or PC O-36:5a | 766.5748 | 8.57 |
| PC P-36:4b or PC O-36:5b | 766.5739 | 9.01 |
| PC P-36:2 or PC O-36:3 | 770.6066 | 9.25 |
| PC P-36:1 or PC O-36:2 | 772.6222 | 9.25 |
| PC P-36:0 or PC O-36:1 | 774.6360 | 9.54 |
| PC P-38:6 or PC O-38:7 | 790.5751 | 8.74 |
| PC P-38:5 or PC O-38:6 | 792.5897 | 8.93 |
| PC P-38:3 or PC O-38:4 | 796.6226 | 9.29 |
| PC P-40:6 or PC O-40:7 | 818.6054 | 9.18 |
| PE 32:0 | 692.5238 | 7.94 |
| PE 34:2 | 716.5226 | 8.51 |
| PE 34:0 | 720.5547 | 8.43 |
| PE 36:4 | 740.5239 | 8.54 |
| PE 36:3 | 742.5387 | 8.65 |
| PE 36:2 | 744.5552 | 8.97 |
| PE 36:1 | 746.5715 | 9.25 |
| PE 36:0 | 748.5861 | 8.84 |
| PE 38:6 | 764.5233 | 8.47 |
| PE 38:5 | 766.5397 | 8.68 |
| PE 38:4 | 768.5547 | 8.97 |
| PE 38:2 | 772.5851 | 8.74 |
| PE 40:6 | 792.5506 | 8.65 |
| PE P-34:2 or PE O-34:3 | 700.5274 | 8.82 |
| PE P-34:1 or PE O-34:2 | 702.5440 | 9.12 |
| PE P-36:4 or PE O-36:5 | 724.5285 | 8.83 |
| PE P-36:3 or PE O-36:4 | 726.5446 | 8.94 |
| PE P-36:2 or PE O-36:3 | 728.5603 | 9.25 |
| PE P-36:1 or PE O-36:2 | 730.5756 | 9.53 |
| PE P-36:0 or PE O-36:1 | 732.5906 | 9.16 |
| PE P-38:6 or PE O-38:7 | 748.5285 | 8.76 |
| PE P-38:5 or PE O-38:6 | 750.5441 | 8.94 |
| PE P-38:4 or PE O-38:5 | 752.5598 | 9.26 |
| PE P-38:2 or PE O-38:3 | 756.5917 | 9.03 |
| PE P-40:6 or PE O-40:7 | 776.5597 | 9.19 |
| PE P-42:10 or PE O-42:11 | 796.5268 | 9.00 |
| PI 38:4 | 887.5639 | 8.93 |
| PS 34:0 | 764.5493 | 8.24 |
| PS 40:6 | 836.5394 | 8.59 |
| Sphingosine | 300.2901 | 4.17 |
| Palmitoyl-EA | 300.2901 | 5.04 |
| Oleoyl-EA | 326.3054 | 6.22 |
| Cer 18:1;O2/16:0 | 538.5201 | 8.37 |
| Cer 18:1;O2/22:0 | 622.6144 | 9.73 |
| Cer 18:1;O2/24:0 | 650.6458 | 10.13 |
| Cer 18:1;O2/24:1 | 648.6305 | 9.88 |
| SM 18:1;O2/14:0 | 675.5447 | 7.72 |
| SM 18:1;O2/16:1 | 701.5592 | 8.09 |
| SM 18:1;O2/16:0 | 703.5755 | 8.23 |
| SM 18:1;O2/18:2 | 749.5578 | 8.07 |
| SM 18:1;O2/18:1 | 729.5915 | 8.40 |
| SM 18:1;O2/18:0 | 731.6070 | 8.72 |
| SM 18:1;O2/20:0 | 759.6375 | 9.44 |
| SM 18:1;O2/22:1 | 785.6531 | 9.54 |
| SM 18:1;O2/22:0 | 787.6690 | 9.62 |
| SM 18:1;O2/24:1 | 813.6849 | 9.66 |
| SM 18:1;O2/24:0 | 815.7009 | 10.02 |
| CE 14:0 | 619.5431 | 11.59 |
| CE 16:1 | 645.5586 | 11.69 |
| CE 16:0 | 647.5745 | 11.98 |
| CE 18:3 | 669.5587 | 11.53 |
| CE 18:2 | 671.5741 | 11.76 |
| CE 18:1 | 673.5900 | 12.08 |
| CE 18:0 | 675.6060 | 12.42 |
| CE 20:5 | 693.5585 | 11.40 |
| CE 20:4 | 695.5741 | 11.64 |
| CE 20:3 | 697.5899 | 11.88 |
| CE 22:6 | 719.5743 | 11.50 |
| CE 22:5 | 721.5898 | 11.71 |
| CE 22:4 | 723.6061 | 12.15 |
| MG 16:1 | 339.2511 | 4.97 |
| MG 18:0 | 381.2977 | 5.88 |
| DG 30:0 | 563.4653 | 8.75 |
| DG 32:2 | 587.4647 | 8.57 |
| DG 32:1 | 589.4810 | 8.88 |
| DG 32:0 | 591.4967 | 9.17 |
| DG 34:3 | 613.4806 | 8.72 |
| DG 34:2 | 615.4964 | 9.03 |
| DG 34:1 | 617.5124 | 9.31 |
| DG 34:0 | 619.5276 | 9.58 |
| DG 36:4 | 639.4966 | 8.86 |
| DG 36:3 | 641.5122 | 9.16 |
| DG 36:2 | 643.5281 | 9.44 |
| DG 36:1 | 645.5438 | 9.72 |
| DG 36:0 | 647.5588 | 9.97 |
| DG 38:5 | 665.5122 | 9.14 |
| DG 38:4 | 667.5284 | 9.42 |
| TG 41:0 | 731.6184 | 10.89 |
| TG 42:0 | 745.6331 | 10.94 |
| TG 43:2 | 755.6188 | 10.75 |
| TG 43:1 | 757.6335 | 10.89 |
| TG 43:0 | 759.6486 | 11.09 |
| TG 44:2 | 769.6331 | 10.79 |
| TG 44:1 | 771.6487 | 11.05 |
| TG 44:0 | 773.6639 | 11.26 |
| TG 45:3 | 781.6317 | 11.03 |
| TG 45:2 | 783.6489 | 11.00 |
| TG 45:1 | 785.6642 | 11.20 |
| TG 45:0 | 787.6801 | 11.54 |
| TG 46:4 | 793.6332 | 10.65 |
| TG 46:3 | 795.6487 | 10.90 |
| TG 46:2 | 797.6638 | 11.12 |
| TG 46:1 | 799.6794 | 11.35 |
| TG 46:0 | 801.6954 | 11.56 |
| TG 47:2 | 811.6796 | 11.28 |
| TG 47:1 | 813.6954 | 11.49 |
| TG 47:0 | 810.7552 | 11.71 |
| TG 48:5 | 819.6479 | 10.87 |
| TG 48:4 | 821.6641 | 10.98 |
| TG 48:3 | 823.6794 | 11.21 |
| TG 48:2 | 825.6941 | 11.43 |
| TG 48:1 | 827.7104 | 11.66 |
| TG 48:0 | 829.7260 | 11.88 |
| TG 49:3 | 837.6957 | 11.36 |
| TG 49:2 | 839.7115 | 11.58 |
| TG 49:1 | 841.7278 | 11.94 |
| TG 49:0 | 838.7886 | 12.04 |
| TG 50:6 | 845.6642 | 11.00 |
| TG 50:5 | 847.6791 | 11.12 |
| TG 50:4 | 849.6942 | 11.52 |
| TG 50:3 | 851.7098 | 11.51 |
| TG 50:2 | 853.7260 | 11.73 |
| TG 50:1 | 855.7418 | 11.97 |
| TG 50:0 | 857.7581 | 12.22 |
| TG 51:3 | 865.7262 | 11.67 |
| TG 51:2 | 867.7419 | 11.90 |
| TG 51:1 | 869.7582 | 12.13 |
| TG 51:0 | 871.7738 | 12.37 |
| TG 52:7 | 871.6801 | 11.10 |
| TG 52:6 | 873.6951 | 11.27 |
| TG 52:5 | 875.7099 | 11.61 |
| TG 52:4 | 877.7253 | 11.59 |
| TG 52:3 | 879.7416 | 11.83 |
| TG 52:2 | 881.7574 | 12.06 |
| TG 52:1 | 883.7736 | 12.31 |
| TG 52:0 | 885.7897 | 12.57 |
| TG 53:3 | 893.7577 | 12.00 |
| TG 53:2 | 895.7738 | 12.23 |
| TG 54:10 | 893.6624 | 10.03 |
| TG 54:9 | 895.6807 | 11.01 |
| TG 54:8 | 897.6957 | 11.18 |
| TG 54:7 | 899.7105 | 11.36 |
| TG 54:6 | 901.7246 | 11.47 |
| TG 54:5 | 903.7412 | 12.03 |
| TG 54:4 | 905.7572 | 11.92 |
| TG 54:3 | 907.7732 | 12.16 |
| TG 54:2 | 909.7893 | 12.40 |
| TG 54:1 | 911.8057 | 12.67 |
| TG 55:3 | 921.7896 | 12.33 |
| TG 55:2 | 923.8059 | 12.58 |
| TG 56:10 | 921.6962 | 11.12 |
| TG 56:9 | 923.7109 | 11.27 |
| TG 56:8 | 925.7256 | 11.48 |
| TG 56:7 | 927.7417 | 11.70 |
| TG 56:6 | 929.7571 | 11.88 |
| TG 56:5 | 931.7733 | 12.12 |
| TG 56:4 | 933.7890 | 12.25 |
| TG 56:3 | 935.8054 | 12.49 |
| TG 56:2 | 937.8212 | 12.77 |
| TG 56:1 | 939.8378 | 13.07 |
| TG 58:11 | 947.7112 | 11.20 |
| TG 58:10 | 949.7257 | 11.35 |
| TG 58:9 | 951.7407 | 11.56 |
| TG 58:8 | 953.7580 | 11.80 |
| TG 58:7 | 955.7733 | 12.01 |
| TG 58:6 | 957.7882 | 12.52 |
| TG 60:12 | 973.7267 | 11.36 |
| Cholesterol | 369.3524 | 7.41 |
| Campesterol | 401.3796 | 7.55 |
| Piperine | 286.1442 | 3.71 |
| Coenzyme Q10 | 885.6736 | 11.09 |
